# Book Review

**Published:** 1926

**Authors:** 


### Reviews of Books.

**Brain and Heart.** By Giulio Fano. Translated by Helen Ingleby with a Foreword by Prof. E. H. Starling. Pp. xv., 142. Humphrey Milford, Oxford University Press. 1926. Price 8s. 6d. net.—Mr. Milford is to be congratulated on publishing these lectures of the great Italian physiologist, since, as Professor Starling says in his Foreword, they represent an epitome of the life work of a very great master, and Professor Fano is to be congratulated that this brief survey of his work has been presented to English readers by such an admirable translation, which preserves the freshness and charm of the original and at the same time presents the subject-matter so easily and clearly. The first two lectures are on "the so-called living matter." After reviewing the recent advances in physics and chemistry in explaining many processes which we observe in living matter, he scouts the idea that these bring us any nearer the understanding of life. Living matter is merely the form taken by the life force, and as such is relatively unimportant. Almost, but perhaps not quite, he persuadeth us to believe that Bergson has said the last word in philosophy. In the next two lectures he describes experiments which show that the whole hierarchical arrangement of the nervous system subserves a series of superimposed inhibitions, till finally the discriminative will of man is made possible by inhibitions of inhibitions in lower levels of function. In the last two lectures, on excitability and automatism, he describes his experiments on the excised hearts of animals and embryos, which show that as we extend the scale in the history of the race the individual automatism gives place to excitability in inverse proportion, and how in the case of the heart the high automatism and low excitability of the auricular end and the high excitability and low automatism of the ventricular end explain most of the modern problems of cardiology.

**Surgery of Childhood.** By John Fraser, M.C., M.D., Ch.M., F.R.C.S. (Eng.). 2 vols. Pp. viii., 604 ; iii., 609–1152. London : Arnold & Co. 1926.—The surgery of childhood differs so materially from that of adults that the appearance of these volumes by Professor Fraser will be welcomed by all those whose work lies to any extent in the treatment of children. Taken as a whole, the construction of the work leaves little to be desired. Some of the rarer deformities might well have been relegated to text books of orthopædics, and some portions (notably the surgery of the spleen) could have been amplified with advantage. Naturally a good deal of the book deals with orthopædic surgery, which forms so large a part of the work of any children's hospital, but the author has selected his material with care, and summarised judiciously. The chapters dealing with surgical tuberculosis are excellent, and the article on the making of celluloid splints could hardly be improved upon. In Volume I. the description of the various methods for the treatment of burns is particularly valuable. The book is thoroughly up to date and includes a good deal of recent work. Printing, illustrations and index are good, and there are unusually few typographical errors. Any surgeon would find it quite a sound investment.

**Tuberculous Disease of the Hip Joint.** By George Perkins, F.R.C.S., Assistant Surgeon to the Royal National Orthopædic Hospital. Pp. 118, figures 32. Humphrey Milford, Oxford University Press. Price 6s.—This, the first "Robert Jones" prize monograph, is a clear and simple account of hip disease. The diagnosis of only the early stages is considered and a number of most excellent X-ray pictures greatly help the text. A follow-up of 50 cases with description of late results as related to the type of disease and method of treatment is the most valuable feature of the book.

**Radiotherapy in Relation to General Medicine.** By F. Hernaman-Johnson, M.D. Pp. viii., 211. Humphrey Milford, Oxford University Press. 1926. Price 5s. net.—This little book, essentially non-technical, is alive with sane and suggestive thought upon the subject of Radiant Energy and its uses and limitations in the art of Therapeutics. The title adequately expresses its scope, and it should do much to counteract extreme and uninformed views about this rather difficult subject, and at the same time stimulate a real interest in the great and still relatively unexplored possibilities of Radiotherapy in relation to general medicine. Rarely has one come across a book of this type which can be so heartily recommended, both to the general practitioner or surgeon and to the specialist.

When one reflects that matter is now considered as a form of energy, that life in all its forms is a manifestation of energy, and that the cause of its evolution has been largely determined by Radiant Energy—life has been bathed in various forms of Radiant Energy since its birth—it becomes obvious that in artificially-produced radiations we have a most potent addition to our therapeutic armamentarium, the intelligent study of which becomes almost a duty, when one considers the present state of medical science. The author, after reviewing in a most interesting manner the physics and biological effects of Radiation, discusses at some length the general nature of disease and what we can hope for in the action of remedies—this chapter might have been condensed somewhat—and proceeds to bring Radiation-Therapy into line with other forms of treatment. The latter half of the book deals more specifically with his experiences and suggestions in dealing with the Cancer problem, and the treatment of various infective and endocrine disturbances, and he ends with a strong plea for an intelligent combined attack on disease, using such therapeutic agents as we now have at our command, not as a blunderbuss, but in proper and rational combination, and directed by specialists, who are also " general practitioners " in their medical outlook, working in co-operation.

**The Medical Annual.** 44th Year. Pp. 616. Bristol: John Wright & Sons Ltd. 1926. Price 20s. net.—The great value of the *Annual*, now in its forty-fourth year, is so universally recognised that it needs no recommendation here. The present issue is an excellent one, but many who esteem its invaluable accounts of recent improvements in treatment, diagnosis and prevention of disease seem to be ignorant of its " special guide " sections—its lists of recent and new books, classified, and giving the prices and publishers, those of institutions, homes and sanatoria, of medical and scientific societies and periodicals, of Life Assurance Offices, of manufacturers of new appliances, of many official boards (by the way, we miss the Board of Control), such as the personnel of the General Medical Council and the Ministry of Health. Now these are of the greatest use to the busy practitioner and add much to the value of the book. Then, too, there are indexes, published separately every ten years, helpful for those who have access to the back volumes. In this way anyone working up a subject thoroughly can find references to and abstracts of most of the important papers on his subject in

English journals during recent years. In no other way can he so quickly get an outline of what has been done and its results. The aid thus given by the *Annual* to scientific study is unique and of national importance. We are anxious to draw attention to these features of the book, though the very interesting details in the present volume of recent advances in medicine and surgery might well take up our space.

**Favourite Prescriptions.** By Espine Ward, M.D. (Belfast). Pp. 96. London: J. & A. Churchill. 1926. Price 5s.—This is a small book which contains a large number of prescriptions which should be useful to the newly-qualified practitioner, but a distinct danger in the hands of the more ignorant general public. Its value is greatly diminished in that these prescriptions are arranged under the headings of definite diseases, and not under the headings of the principal drugs prescribed. The following are examples of the danger attending the course adopted by the author: There is only one prescription for such diseases as Pneumonia and Bright's Disease in its many forms. Under the head of Epilepsy we find three prescriptions containing bromides, but no reference is made to the value of luminal in these cases. It would appear to be rather dangerous to suggest arsenic as the proper treatment for eczema in all its protean forms, and still more dangerous to prescribe sulphonal in small repeated doses for mania. In spite of the above criticisms, there are many prescriptions which should be useful and suggestive to the busy practitioner. The hints for the treatment of poisoning at the beginning of the book are handy, concise and valuable.

